# Applying clinical guidelines in general practice: a qualitative study of potential complications

**DOI:** 10.1186/s12875-016-0490-3

**Published:** 2016-07-22

**Authors:** Bjarne Austad, Irene Hetlevik, Bente Prytz Mjølstad, Anne-Sofie Helvik

**Affiliations:** Sjøsiden Medical Centre, Trondheim, Norway; General Practice Research Unit, Department of Public Health and General Practice, NTNU, Norwegian University of Science and Technology, PO Box 8905, MTFS, 7491 Trondheim, Norway; Saksvik Medical Centre, Hundhamaren, Norway; Department of Ear, Nose and Throat, Head and Neck Surgery, Trondheim University Hospital, Trondheim, Norway

**Keywords:** General practitioners, Clinical practice guidelines, Guideline adherence, Multimorbidity, Overtreatment, Patient-centered care, Polypharmacy, Qualitative research, Focus groups

## Abstract

**Background:**

Clinical guidelines for single diseases often pose problems in general practice work with multimorbid patients. However, little research focuses on *how* general practice is affected by the demand to follow multiple guidelines. This study explored Norwegian general practitioners’ (GPs’) experiences with and reflections upon the consequences for general practice of applying multiple guidelines.

**Methods:**

Qualitative focus group study carried out in Mid-Norway. The study involved a purposeful sample of 25 Norwegian GPs from four pre-existing groups. Interviews were audio-recorded, transcribed and analyzed using systematic text condensation, i.e. applying a phenomenological approach.

**Results:**

The GPs’ responses clustered around two major topics: 1) Complications for the GPs of applying multiple guidelines; and, 2) Complications for their patients when GPs apply multiple guidelines. For the GPs, applying multiple guidelines created a highly problematic situation as they felt obliged to implement guidelines that were not suited to their patients: too often, the map and the terrain did not match. They also experienced greater insecurity regarding their own practice which, they admitted, resulted in an increased tendency to practice ‘defensive medicine’. For their patients, the GPs experienced that applying multiple guidelines increased the risk of polypharmacy, excessive non-pharmacological recommendations, a tendency toward medicalization and, for some, a reduction in quality of life.

**Conclusions:**

The GPs experienced negative consequences when obliged to apply a variety of single disease guidelines to multimorbid patients, including increased risk of polypharmacy and overtreatment. We believe patient-centered care and the GPs’ courage to non-comply when necessary may aid in reducing these risks. Health care authorities and guideline developers need to be aware of the potential negative effects of applying a single disease focus in general practice, where multimorbidity is highly prevalent.

## Background

General practitioners (GPs) provide care for any health problems patients might have and general practice is regarded as a cornerstone of the health care systems of many countries. Clinical guidelines build on Evidence-Based Medicine (EBM) and are designed to improve the quality of health care and reduce unwanted variations [1]. If GPs do not follow guidelines as delineated, treatments proven by research to be effective will not benefit the population at large, thus posing a challenge both to society and health authorities. The Directorate of Health, the executive agency in Norway tasked with formulating national clinical guidelines, categorizes their recommendations primarily according to the GRADE system [2].

It is well known that adherence to clinical guidelines in general practice is low [3]; most clinical guidelines are designed for the treatment of single diseases while an increasing amount of research has documented that guidelines for single diseases are of little use in the treatment of patients with multimorbidity [4, 5]. Multimorbidity is frequently encountered in general practice, affecting as many as 23 % of the Scottish [6] and 42 % of the adult Norwegian populations [7]. Treating multimorbid patients involves meeting a variety of challenges, only one of which is that guidelines have been created for the treatment of single diseases [8, 9].

Overtreatment is defined as unnecessary health care and has been shown to be highly problematic, both for multimorbid patients and for patients with single, longstanding conditions or risk factors [10, 11]. The reasons for overtreatment seem multifactorial and complex. Questions arise as to the extent to which multiple guidelines are drivers of overtreatment [12]. Boyd et al. documented that adherence to all guidelines simultaneously for a hypothetical multimorbid 79-year-old woman with five different chronic conditions would result in the prescribing of 12 different medications as well as the recommending of a complex, non-pharmacological regimen [13].

In a previous paper, we documented that GPs offered compelling reasons for low adherence to clinical guidelines, despite considering them necessary [14]. One of the main explanations was the mismatch they experienced when caring for the whole patient while using guidelines focused on single diseases [14]. Caring for the whole person rather than just the single disease is a well-known characteristic of general practice [15]. Nonetheless, GPs are expected to implement a variety of clinical guidelines simultaneously, each of which was designed for the treatment of a single disease. Adherence to guidelines is mandated by medical regulations in Norway [16]. The failure to follow guidelines for each single disease has sometimes resulted in practitioners’ work being subjected to professional review.

Despite the gap between clinical practice and guidelines being well known, little research focuses on *how* general practice is affected by the demand to follow multiple guidelines [17]. The aim of this study was to explore Norwegian GPs’ experiences with and reflections upon the consequences of guidelines for themselves and their patients, particularly multiple guidelines each designed for the treatment of a single disease.

## Methods

### Research design, recruitment and sampling

We chose a qualitative design as this is regarded as the best way to explore and provide rich descriptions of a complex phenomenon [18, 19]. The theoretical framework we used is phenomenology, a philosophy and methodology that relies on first-person accounts as a source of knowledge [20, 21]. We chose to hold focus group interviews with pre-existing GP groups under the assumption that their familiarity with each other would allow the participants to reflect more openly [22]. The Norwegian Continuing Medical Education (CME) organizes groups of GPs who are working towards fulfilling the mandatory requirements of specialist training in general practice (junior groups) and registers self-selected groups whose members have already completed their specialization (senior groups) [23]. In Norway, participation in a senior group is a requirement for maintaining one’s status as specialist. Utilizing the CME system allowed us to have an overview of the existing local groups that we could approach. For reasons of convenience, we invited groups from only one region of the country, Mid-Norway, to participate. To ensure a strategic, purposeful sample of GPs with a spread of age and work experience, we approached two junior groups and two senior groups and planned to include more groups if the material was not saturated. All four groups agreed to participate.

### Interview settings

In 2013, each group was interviewed once at the location where they usually met. Three groups met at medical centers while one met at another meeting room. The interviews lasted 60–90 min. Two researchers participated in all the interviews, one as a moderator and the other as an assistant. The moderator (BA) ensured that all participants participated in the discussion and also facilitated the elaboration of their varying opinions and views. As well as posing some questions, the assistant (BPM or HTB – see Acknowledgments) was responsible for the audio-recordings and the notation of the order of speech.

The interviews started with the moderator reading from a Norwegian article that problematized applying disease-specific clinical guidelines in the treatment of multimorbid and elderly patients in general practice [24]. The groups were asked what they thought about the article and whether it was recognizable from their clinical practices. The interview guide included the following main themes: 1) use of clinical guidelines in their daily practice; 2) use of clinical guidelines with multimorbid patients; 3) guideline characteristics that might facilitate or hinder GPs’ adherence; and, 4) guidelines as quality assurance in clinical practice. The questions were open-ended and the order flexible. Topics concerning the complications created for general practice by applying multiple clinical guidelines arose spontaneously during all the interviews and were then further explored. The group interviews were audio-recorded and transcribed verbatim.

### Analysis and interpretation

To analyze the data, we used ‘systematic text condensation’, a thematic cross-case analysis based on Giorgi’s phenomenological analysis [25, 26]. It consisted of the following steps: 1) reading and listening to all the material and obtaining an overall impression; 2) identifying ‘meaning units’, units of text providing knowledge of the phenomenon being studied, and then sorting and coding them; 3) condensing and abstracting the meaning within each of the coded groups; and, 4) synthesizing the condensations into major topics and sub-topics that reflected the GPs’ experiences of how following multiple clinical guidelines affected general practice. All the authors participated in the analysis and interpretation of the data. All the authors have clinical experience as either GPs (BA, BPM, and IH) or as a nurse (ASH), and all four are also university researchers and educators.

## Results

Participant characteristics are listed in Table 1. While our aim was to explore the *consequences* of applying multiple guidelines, the GPs’ interview responses to our open-ended questions clustered spontaneously around *complications.* We categorized the results into: 1) Complications for the GPs of applying multiple guidelines; and, 2) Complications for their patients when GPs apply multiple guidelines. We sub-divided those two topics into the sub-topics elaborated below.

**Table 1:** Characteristics of the study participants

	Group 1 (*n* = 7)	Group 2 (*n* = 8)	Group 3 (*n* = 3)	Group 4 (*n* = 7)	Total (*n* = 25)
Female (*n*)	3	2	0	5	10
Age in years min – max (mean)	31 – 39 (34.3)	45 – 62 (55.9)	40 – 47 (44.3)	31 – 45 (37.0)	31 – 62 (43.4)
Years as GP^a^ min – max (mean)	1 – 4 (2.9)^a^	8 – 35 (22.6)	12 – 13 (12.3)	0 – 4 (2.4)^a^	0 – 35 (9.6)
Specialist in general practice (*n*)	0	8	3	0	11
Specialist in another medical discipline (*n*)	1	1	0	1	3

^a^Years of experience in open, unselected general practice. Two of the participants with the least experience as GPs had 5–6 years of experience in an Emergency Ward

### Complications for the GPs of applying multiple guidelines

#### A highly problematic situation

Some guidelines were experienced as contributing to safety and aiding the GPs in choosing treatments. Nonetheless, attempts to adhere to the combined total of all applicable clinical guidelines resulted in the GPs feeling they lost the overview over the relevant recommendations, which in turn increased their frustration and a tendency to give up on guidelines altogether. The GPs experienced the situation to be highly problematic. As one GP said:

*When you have so many chronic diseases and are expected to follow all the guidelines – the result is chaos. (Group 1, M4)*

They asserted that, despite clinical guidelines designed for the treatment of single diseases being of little value when treating multimorbid patients, the GPs still felt themselves to be under pressure to attempt to adhere to all of them – even when, as they put it, the map and the terrain simply did not match. In the following quote, one GP reflects over the shortcomings of clinical guidelines in relation to complex medical histories.

*There are no guidelines yet which can encompass ‘complexity-based medicine’. To grasp how to work with the complexity we confront as GPs requires a massive, theoretical quantum leap. Perhaps in 10–15 years we will realize that all of today’s reductionist guidelines within the natural sciences were wrong and had led us astray. (Group 2, M7)*

### Increased insecurity

Some GPs experienced a growing insecurity as to whether or not their own clinical practice was in accordance with the guidelines. One claimed that if someone were to look systematically at perhaps 100 patient records from each of the GPs in the focus group, mistakes would probably be found in all of them. The total number of demands in the guidelines was simply impossible to meet. This created insecurity.

*The insecurity that a ‘guideline hell’ brings is negative, but that is not talked about very often. (Group 2, M7)*

Some of the senior GPs did not feel less secure. One, who was close to retirement age, said that he did not worry anymore about any professional review procedures. However, regardless of how long they had been in practice, most of the GPs hoped to avoid being subjected to licensing review. They feared that the monitoring authorities would evaluate their work based solely on what they should have done according to existing guidelines, without taking their clinical judgement into consideration.

### More ‘defensive medicine’

The fear of criticism or of being subjected to professional review for failing to adhere to guidelines seems to have led to GPs practicing more ‘defensive medicine’, such as increasing their prescribing of drugs and making more referrals to specialists than they actually thought were necessary. As one GP put it:

*I often chose to ‘protect my back’ by doing too much, by following up too thoroughly, for instance, ordering additional x-rays or other extra examinations. (Group 4, K10)*

Another participant pointed out that GPs are rarely if ever subjected to professional review for overtreatment.

*We never get criticized for doing too much. You don’t get in trouble for having initiated unnecessary examinations even if they lead to complications. But you can be sure you’ll get in trouble if you haven’t done enough! We’re much more vulnerable to the entire health care system’s expectation that things must be done. There’s an intense* ‘*action imperative*’ *to do more. (Group 2, M7)*

Keeping thorough patient records seems to be another way the GPs guarded themselves, especially when they knew they had deviated from the guidelines.

*When I deviate from the guidelines, I am careful to write my reasons down in the patient record. For instance, if I take a patient off acetylic acid because he developed a stomach ulcer, I write that I am aware of the increased risk of a blood clot. Good record-keeping helps protect me. (Group 3, M11)*

### Complications for their patients when GPs apply multiple guidelines

#### Excessive pharmacological and non-pharmacological treatment

Most guidelines include recommendations for medical treatments for diseases and risk factors. The GPs claimed polypharmacy to be a widespread problem for many of their patients, especially the elderly and multimorbid, and they worried that adhering to multiple guidelines might exacerbate that tendency.

*It’s great that there are guidelines, and I try to follow them. But when the patients have several diseases, there are too many guideline recommendations. Especially when patients are getting older, how much medicine should you give them? (Group 3, M12)*

Polypharmacy was considered problematic as it could result in side effects and/or drug interactions while the actual benefit to the individual patient might remain questionable. One participant stated that GPs have a responsibility to counteract polypharmacy. However, several reported difficulty discontinuing medications, especially if a specialist had initiated the treatment.

*I see how patients go into the hospital and have new medications added because the hospital has followed the guidelines. We often have to take responsibility later for having the patients discontinue some meds and we thereby ‘break the rules’. That’s no easy job! But we have to try to see the whole patient. (Group 4, K9)*

Some guidelines include non-pharmacological recommendations. The GPs experienced that some of these proved too time-consuming to follow up on for many of the patients – in some cases, even completely unrealistic. As a result, the GPs tried to individualize the recommendations, to tailor them to the patients’ needs rather than adhere to them exactly as stated.

*The treatment must be planned, individually, based on the patient's functional ability, interests, what he actually manages to follow up on in everyday life, how many activities he can tolerate during a week. The non-pharmacological regimen should not place an additional burden on people already struggling with chronic diseases. (Group 4, K6)*

### Increased medicalization

The existing guidelines refer to criteria for disease definition, some of which have changed over time. The GPs had experienced treating several patients diagnosed with diabetes and hypertension after such changes of definitions were made. They also described a growing trend wherein complaints previously considered to be common ailments might now be regarded as diseases that physicians were obligated to treat. They were concerned that an increased tendency toward medicalization might result from increasing the percentage of the population that multiple guidelines now defined as being at risk.

*It seems to me as if some of the guidelines’ recommendations are implying: Everybody needs treatment, but so many people just don’t know it yet. We GPs have to counteract this and let our patients know that we don’t think they’ll live any longer or have a better life if we just put them on one additional drug. (Group 2, M6)*

### Reduced quality of life

The GPs shared stories about overly-extensive pharmacological and/or non-pharmacological treatments having contributed to a reduction in quality of life for some of their multimorbid patients. Even though longstanding chronic diseases were considered important to treat and follow up, dilemmas arose when guidelines recommended treatments that the GPs meant did not benefit the patients’ overall situation or quality of life.

*A patient of mine with atrial fibrillation, COPD and heart failure is often hospitalized because of dizziness. The cardiologists treat him every time with a beta blocker, in accordance with the guidelines, but he gets bradycard, so I deprescribe it after every hospital stay. Seen in isolation, he could conceivably benefit from being on that medication, but he does not tolerate it. I regulate treatment according to the patient’s symptoms and overall situation. (Group 4, M14)*

In addition, the GPs experienced that the guidelines did not take into account their patients’ varying attitudes towards treatment and taking medications.

*What matters most is the patients’ quality of life. We as GPs have to listen to what the patients say, and do the best we can to relieve their suffering. (Group 3, M13)*

## Discussion

### Summary of the main findings

For the GPs, the obligation to apply multiple guidelines each of which was designed to treat single diseases created various complications. They found it highly problematic to be required to implement guidelines that did not fit their patients, when the map and the terrain simply did not match. They also experienced greater insecurity about their own practice which, they admitted, increased the tendency to practice ‘defensive medicine’. The complications for their patients which the GPs experienced when applying multiple guidelines included an increased risk of polypharmacy, of excessive non-pharmacological recommendations, an increased tendency toward medicalization and a potentially reduced quality of life.

### Strengths and limitations of the study

Diversity is considered a strength in qualitative studies [27]. Although all participants worked in Mid-Norway (Table 1), our sample of 25 GPs was diverse as regards work experience as well as demographic variables such as age and gender. Otherwise, the participants did not differ systematically from Norwegian GPs as a group [28]. When we realized shortly before one of the focus group sessions that only three participants would be available to attend, we considered choosing a different group. We decided not to cancel the interview and, despite the small number of participants, the discussion that ensued proved to be so rich that we included the data in our material. After conducting four focus groups, we carried out critical readings of the transcripts and determined that the material was sufficiently saturated. As we only interviewed the GPs, all the descriptions of the complications for their patients of following multiple guidelines were from the GPs’ point of view, not that of the patients themselves. Nevertheless, we consider the GPs’ perceptions to be reliable since they work closely with their patients and are trained to observe how their patients react to medical advice and treatment.

The study was conducted in Norway where national clinical guidelines are provided by the health authorities and adherence is regulated. This may limit how transferable some of our findings might be to countries following different approaches to the development, implementation and regulation of clinical guidelines.

The fact that the moderator was a GP can be considered both as a strength and a limitation. Talking to a member of their own profession and presuming a common understanding of clinical work may have helped the participants speak more openly. On the other hand, the participants might have wanted to ‘comfort’ the moderator, and consequently downplayed important contradictory views or nuances. To address this potential limitation, we attempted to make our preconceptions overt, to ask open-ended questions and encourage contradicting views. All authors also evaluated the interview guide and the results critically. Our experience was that the moderator being a GP facilitated the disclosure of whatever disagreements existed among the participants.

We began each of the interviews by reading from an article that we presumed would awaken the GPs’ awareness of their experiences with adhering to multiple guidelines and stimulate them to reflect on the consequences [24]. The article did not seem to arouse controversy; most of the GPs recognized the patient story in it from their own practice. Conceivably, this way of opening the focus group interviews may have influenced the participants to respond more critically to the consequences of multiple guidelines than they actually were. However, we think the participants’ familiarity with each other helped them to feel safe enough to disagree, both with the article and with each other. This added variety and enriched the complexity of our material.

### Implications of the findings in context of existing research

#### Guidelines as drivers of overtreatment

In recent years, the international focus on overtreatment and overdiagnosis has increased, especially concerning multimorbid and elderly patients [29, 30]. The British Medical Journal’s series entitled, “Too Much Medicine”, and The Journal of the American Medical Association’s, “Less is More”, are examples of this increased focus [31, 32]. The following statement was made at the 2013 international scientific conference, ‘Preventing Overdiagnosis’: “Overdiagnosis harms people worldwide and exacerbates undertreatment by wasting much needed resources” [33]. Still, the definition of overdiagnosis is not clear and the controversy regarding the extent of the problem continues [34]. The GPs in the present study expressed that, despite their intent to avoid overtreatment, polypharmacy and the recommendation of more treatment than they actually deemed necessary, these tendencies represented a widespread problem for their patients. This would indicate that overtreatment is a challenge for Norwegian general practice.

Overtreatment seems to be multifactorial and complex, and several drivers have been identified [35, 36]. One of the drivers which we identified in our interviews with the GPs was the obligation to implement multiple guidelines each designed to treat single diseases when treating multimorbid patients and patients with a variety of risk factors. This finding is supported in other studies criticizing clinical guidelines for extending disease definitions and thus introducing treatment to a larger segment of the population [37, 38].

At the same time, the expressed intention of clinical guidelines is not to provoke overtreatment but to help in offering patients the best treatment possible. Some guidelines include recommendations for when to refrain from offering treatment; others state specifically that guidelines are only to be considered supplementary to clinical judgement [39, 40]. Also, some multimorbid patients need several medications [41]. However, in our findings, GPs’ expressed concern about the need to safeguard themselves legally, prescribing medication in order to ‘cover their back’ rather than because they considered it medically necessary for the patient. This indicates how difficult the pressure to adhere to guidelines can be to manage in actual practice. This finding is supported by an article in the BMJ that questions whether we have given guidelines too much power [42]. Also, the fact that the Norwegian health authorities expect GPs to follow national guidelines might increase the pressure to adhere to multiple guidelines simultaneously, and thereby contribute to overtreatment [16].

### Evidence-based medicine in general practice

Clinical guidelines build on EBM and are most often designed to treat single diseases or risk factors [43]. Documentation of the effectiveness of prescribed medication is essential also within general practice. Problems arise, however, and the complexity increases for both the GP and the patient, when several treatments are applied to the same person [44]. There is very limited empirical evidence regarding the effects of mixing medications since they are usually studied one at a time. It is well known that a single disease focus does not seem to function as intended in primary care [45, 46]. The reasons for this have been highly debated with both too much and too little application of EBM being criticized [47]. A literature review of NICE guidelines relating to primary care documented that nearly two-thirds of the publications cited were of uncertain relevance to patients in primary care, and some have claimed EBM to be a movement in crisis [48, 49]. Questions have also been raised as to whether the lacking success of guidelines is implicit within the traditional, biomedical model in which people are treated as if they were advanced, biological clock-works [50, 51].

One Irish study documented that GPs make compromises between patient-centered care and care based on EBM in the management of multimorbid patients [52]. In our study, the GPs expressed that the obligation to implement multiple guidelines designed for treating single diseases that did not benefit the patients’ overall health or quality of life left them in a highly problematic and chaotic situation. Focus on the whole patient rather than single diseases is a well-known characteristic of general practice. In their definition of general practice, the European section of the World Organization of Family Doctors (Wonca Europe) states that patient-centered care is a key feature [53]. One of Barbara Starfield’s four main features of primary care was: “long-term person- (not disease) focused care” [54]. The patient-centered model, as opposed to the doctor-centered model, ascribes more value to the presented problem of the patient and less to single diseases [17]. This model challenges the disease focus found in clinical guidelines, and thereby also the biomedical research on which the guidelines are based. Working in a patient-centered way in general practice, we believe, can contribute to counteracting some of the tendency toward overtreatment.

## Conclusions

The GPs’ experienced various negative consequences when adhering to multiple guidelines designed to treat single diseases, including their acting as a driver for polypharmacy and overtreatment. Adherence to clinical guidelines for treating single diseases was experienced as incompatible with a patient-centered approach to the treatment of patients with multimorbidity; the map and the terrain did not match.

This study contributes to a critique of the paradigm in which ‘best practice’ is based on clinical guidelines and biomedical research for single diseases or fragments. As long as most of the health care system remains deeply rooted in this paradigm, designing an ‘alternative’ approach will remain a difficult task, one that clearly exceeds the scope of this study. Still, we believe patient-centered care and the GPs’ courage to non-comply when necessary can serve as countermeasures to prevent overtreatment. Health care authorities and guideline developers need to be aware of the potential negative effects of single disease focus in general practice, where multimorbidity is highly prevalent.

## Abbreviations

EBM, evidence-based medicine; GP, general practitioner; GRADE, the grading of recommendations assessment, development and evaluation; NICE, the national institute for health and care excellence.
